# A Cross Talking between the Gut Microbiota and Metabolites of Participants in a Confined Environment

**DOI:** 10.3390/nu16111761

**Published:** 2024-06-04

**Authors:** Xin Song, Ziying Wang, Yongjun Xia, Zheng Chen, Guangqiang Wang, Yijin Yang, Beiwei Zhu, Lianzhong Ai, Haodan Xu, Chuan Wang

**Affiliations:** 1 School of Health Science and Engineering, Shanghai Engineering Research Center of Food Microbiology, University of Shanghai for Science and Technology, Shanghai 200093, China; daohongxuan@126.com (X.S.); dreamup@126.com (Y.X.); 1015wanggq@163.com (G.W.); soliaran@163.com (Y.Y.); ailianzhong1@126.com (L.A.); 2 Naval Medical Center, Naval Medical University, Shanghai 200433, China; wziy0910@hotmail.com; 3 School of Food Science and Technology, Dalian Polytechnic University, Dalian 116034, China; sky_527@163.com (Z.C.); zhubeiwei@163.com (B.Z.)

**Keywords:** confined environment, gut microbiota, non-target metabolome, glycerophospholipid metabolism, correlation analysis

## Abstract

Certain workplaces, like deep-sea voyages, subject workers to chronic psychological stress and circadian rhythm disorders due to confined environments and frequent shifts. In this study, participants lived in a strictly controlled confined environment, and we analyzed the effects of a confined environment on gut microbiota and metabolites. The results showed that living in confined environments can significantly alter both the gut microbiota and the gut metabolome, particularly affecting lipid metabolism pathways like glycerophospholipid metabolism. There was a significant reduction in the abundance of *Faecalibacterium* and *Bacteroides*, while *Blautia*, *Bifidobacterium*, and *Collinsella* showed significant increases. An association analysis revealed a strong correlation between changes in the gut microbiota and the metabolome. Four upregulated lipid metabolites may serve as biomarkers for damage induced by confined environments, and certain gut microbiota alterations, such as those involving *Faecalibacterium* and *Bacteroides*, could be potential psychobiotics or therapeutic targets for enhancing mental health in a confined environment.

## 1. Introduction

Specific working environments like ocean navigation and deep-sea scientific expeditions feature confined spaces for work and living, which negatively impact workers due to factors including noise and circadian rhythm disruptions [1,2]. The small space and social isolation of confined environments lead to a gradual increase in the psychological pressure experienced by workers [3]. Studies have shown that confined environments significantly affect the emotional and physical well-being and work efficiency of individuals. However, the majority of these studies take place in semi-open settings like cargo ships on extended voyages, offering participants more room for movement and the ability to interact with the outside world.

Besides diet and antibiotic use, heightened psychological stress may induce either temporary or permanent alterations in the gut microbiota [4,5]. Individuals working in confined environments are often required to work frequent shifts, disrupting their circadian rhythms and exacerbating emotional and psychological stress, including anxiety and depression. Insufficient sleep and circadian rhythm disorders can significantly disrupt the gut microbiota’s rhythms, impacting immunity, digestion, and behavior [6,7,8]. Research indicates a robust connection between circadian rhythm regulation, gut microbiota, and psychological stress. For instance, chronic stress over time in mice can alter the gut microbiota’s composition and functionality, increase the brain’s production of kynurenine and its metabolites, and disrupt tryptophan metabolism, leading to diminished neurogenesis in the hippocampus and depression [9,10]. In conclusion, chronic stress from environmental and other factors can modify the gut microbiota and impact neurotransmitter metabolism, causing an imbalance in the brain–gut axis’s two-way communication. This may result in depression, irritability, and cognitive decline.

In confined work environments, workers undergo continuous shifts. As a result, their biological rhythms, sleep cycles, and eating habits become misaligned, leading to intestinal dysbacteriosis and changes in intestinal permeability, thereby increasing the risk of metabolic diseases like diabetes and coronary heart disease [11,12,13]. Night-shift work can result in nutritional imbalances and biological clock disruptions, contributing to metabolic syndrome [14]. Moreover, night-shift work may heighten emotional vulnerability, as greater disturbances in circadian rhythms are associated with heightened depression and anxiety-like behaviors [15]. Compared with shift workers in normal environments, those in confined environments experience greater emotional stress. Such stress can induce emotional disturbances that affect workers’ task responsiveness and accuracy, especially those in pivotal roles.

Previous studies on confined working and living environments have focused on improving living conditions, alleviating workers’ anxiety, and increasing their work efficiency through the control of elements like lighting and temperature [16,17,18]. Nevertheless, the specific alterations in gut microbial abundance and metabolite profiles among individuals in confined environments, as well as the relationship between these changes, remain unclear. Consequently, examining the intrinsic connections between gut microbiota and metabolism in individuals living and working in confined environments is essential.

This study simulates a closed underwater environment such as a submarine, where subjects cannot exit the simulation chamber, are unable to communicate with the outside world through mobile phones or computers, and work in frequent shifts according to a work rhythm. The impact of such a strictly confined space on subjects will be more pronounced, especially on the gut microbiota and its metabolites.

Therefore, this manuscript primarily explores the effects of a strictly confined environment (worked in continuous shifts) on the gut microbiota and metabolites in subjects. Diversity sequencing and metabolomics technology were used to analyze the composition of and elucidate the associations between gut microbiota and metabolites and to screen for metabolic markers. By examining the relationship between gut microbiota and distinct metabolites, the goal is to identify potential biomarkers that may be utilized to assess or counteract the impacts of confinement. This study establishes a theoretical basis for future research aimed at enhancing the health and work efficiency of individuals residing and working in confined environments, which will facilitate the formulation of targeted strategies.

## 2. Materials and Methods

### 2.1. Participants

Twelve male participants were selected who did not have cardiovascular, respiratory, and other diseases, maintained a good sleep schedule for 7 days before the trial, and did not consume alcohol, antibiotics, and other drugs. The participants had signed the informed consent form and understood the process and risks of the experiment. All experiments were approved and performed following the guidelines of the Ethical Committee of Naval Medical University (Approval No. 2023032302).

### 2.2. Confined Environment

**Figure 1:**
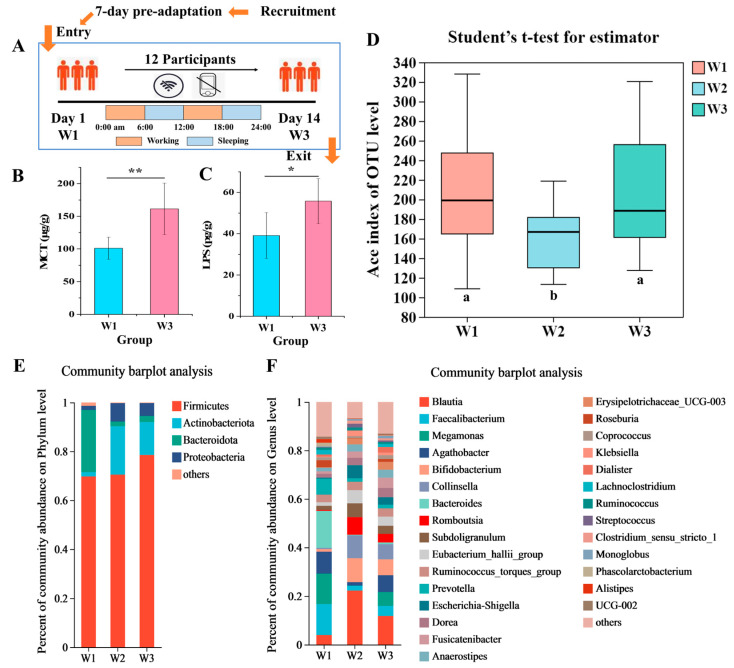
Effect of confined environment on lipopolysaccharides (LPSs), mast cell trypsin (MCT), and intestinal microbiota composition. (**A**) Flow chart of experimental groups; (**B**) Content of MCT in fecal samples. (**C**) Content of LPS in fecal samples. (**D**) α-diversity changes of gut microbiota in different groups. (**E**) Community bar-plot analysis of relative abundance of gut microbiota on phylum level. (**F**) Community bar-plot analysis of relative abundance of gut microbiota on genus level. Results are expressed as the mean ± SEM of participants for each experimental group (*n* = 12). The significance of differences between the data was assessed using one-way ANOVA by Dunnett’s analysis, * *p* < 0.05, ** *p* < 0.01. Different letters (a, b) indicate significant differences among the groups at *p* < 0.05.

This experiment was carried out in a 1:1 restored submarine simulation cabin, which had a normal pressure, room temperature, and closed cabin, an effective volume of 200 m^3^, and the temperature and relative humidity errors in the room were less than 0.5 °C and 5% RH, respectively. During the experiment, participants were allowed to have a regular diet, but alcohol consumption and smoking were strictly prohibited. The participants could use computers, fitness equipment, etc., but there was no external network, and the submarine environment simulation cabin was in a state of information isolation. During the trial, all participants were placed on a strict shift system, working 6 h and resting for 6 h, and the trial duration was 14 days and nights. The flow chart of the experimental groups is shown in Figure 1A. To eliminate the impact caused by dietary differences, after recruitment, participants will be pre-adapted in a normal environment for 7 days. During this period, the diet of the participants will be consistent with that of the confined environment experiment, and they will be free to move and communicate with the outside world. After the adaptation period, participants will enter the confined environment simulation chamber to officially begin the experiment, which will last for 14 days.

### 2.3. Sample Collection

Fecal samples of participants were collected at 18:00–22:00 on days 1, 7, and day 14. The fecal samples of one subject were added into sterilized 10 mL Eppendorf tubes on ice and stored at −80 °C. On the first day after officially entering the simulation chamber, fecal samples from the participants were collected as the control group W1.

### 2.4. DNA Extraction, PCR Amplification, and Illumina MiSeq Sequencing

Fecal DNA was extracted from fecal samples using an E.Z.N.A. stool DNA Kit (Omega Bio-tek, Norcross, GA, USA) following the manufacturer’s protocol. The hypervariable V3-V4 regions of the bacterial 16S rRNA genes were amplified with primers 338F and 806R. PCR amplification of the 16S rRNA gene was performed in triplicate using a mixture containing 10 ng template DNA, 2 μL of 2.5 mM dNTPs, 0.8 μL of each primer at 5 μM, 0.4 μL Fast Pfu polymerase, 4 μL of 5× Fast Pfu Buffer, and ddH_2_O to a final volume of 20 μL. PCR amplification cycling conditions were as follows: initial denaturation at 95 °C for 3 min, followed by 27 cycles at 95 °C for 30 s, 55 °C for 30 s, and extension at 72 °C for 30 s and single extension at 72 °C for 10 min. The quality of PCR products was quantified using QuantiFluor™-ST system (Promega, Madison, WI, USA) in accordance with the standard protocols. Subsequently, purified PCR products were sequenced using an Illumina MiSeq platform (Illumina, San Diego, CA, USA) at Majorbio Bio-Pharm Technology Co., Ltd., Shanghai, China.

### 2.5. Microbiota Analysis

Microbiota analysis of participants was performed by Majorbio Cloud (https://cloud.majorbio.com, accessed on 15 September 2023). All raw reads were demultiplexed and quality-filtered using QIIME (version 1.9.1) with the following criteria: (1) the 300 bp reads were truncated at any site receiving an average quality score < 20 over a 50 bp sliding window; (2) sequences with reads containing ambiguous characters, or two nucleotide mismatches in primer matching were removed. The optimized sequences were clustered into operational taxonomic units (OTUs) using UPARSE (version 11, http://drive5.com/uparse/, accessed on 15 September 2023). RDP Classifier (version 2.13, http://rdp.cme.msu.edu/, accessed on 15 September 2023) was used to analyze the taxonomy of each 16S rRNA gene sequence, and against the SILVA (version 138) 16S rRNA database using a confidence threshold of 70%.

### 2.6. Metabolomic Analysis

Metabolite extraction: Fecal samples of 50 mg were accurately weighed, and the metabolites were extracted using a 400 µL methanol–water (1:1, *v*/*v*) solution and grinding beads. L-2-chlorophenylalanine (0.02 mg/mL) was added as an internal standard. After grinding for 30 s, ultrasonic extraction was performed for 30 min at 5 °C and 40 kHz. The supernatant was collected by centrifugation at 13,000× *g* at 4 °C for 15 min and carefully transferred to sample vials for LC–MS/MS analysis.

UHPLC–MS/MS analysis: UHPLC-Q Exactive HF-X system equipped with an ACQUITY UPLC HSS T3 column (100 mm × 2.1 mm i.d., 1.8 µm; Waters, Milford, MA, USA). The mobile phases were as follows: A, 95% water + 5% acetonitrile (containing 0.1% formic acid); and B, 47.5% acetonitrile + 47.5% isopropanol + 5% water (containing 0.1% formic acid). The column temperature was set at 40 °C. The mass spectrometric data were collected using a Thermo UHPLC-Q Exactive Mass Spectrometer equipped with an electrospray ionization source operating in either positive or negative ion mode. The optimal conditions were set as follows: auxiliary gas heater temperature, 425 °C; sheath gas flow rate, 40 psi; auxiliary gas flow rate, 30 psi; ion-spray voltage floating, −2800 V in negative mode and 3500 V in positive mode; and normalized collision energy, 20–40–60 V rolling for MS/MS. Data acquisition was performed in DDA mode. The detection was carried out over a mass range of 70–1050 *m*/*z*.

Data preprocessing and annotation: After UHPLC–time-of-flight/MS analyses, the raw data were imported into Progenesis QI 2.3 (Nonlinear Dynamics, Waters) for peak detection and alignment. The preprocessing results generated a data matrix that consisted of the retention time, mass-to-charge ratio (*m*/*z*) values, and peak intensity. Metabolic features detected at a frequency of at least 80% in any set of samples were retained. After filtering, the minimum metabolite values were imputed for specific samples in which the metabolite levels fell below the lower limit of quantitation, and each metabolic feature was normalized by summing. The internal standard was used for data quality control (QC, reproducibility). Metabolic features for which the relative standard deviation of the QC values was >30% were discarded. The mass spectra of these metabolic features were identified using the accurate mass, MS/MS fragment spectra, and the isotope ratio difference obtained from reliable biochemical databases, namely the Human Metabolome Database (HMDB, http://www.hmdb.ca/, accessed on 20 September 2023) and the Metlin Database (https://metlin.scripps.edu/, accessed on 20 September 2023). The mass tolerance between the measured *m*/*z* values and the exact mass of the components of interest was ±10 ppm. For metabolites with MS/MS confirmation, only those with an MS/MS fragment score > 30 were considered to be confidently identified. Otherwise, the metabolites had only tentative assignments.

### 2.7. Determination of MCT and LPS in Fecal Samples

After accurate weighing from the sample, 250 mg of stool was diluted in 1.5 mL of precooled phosphate-buffered saline (PBS) with 1 mM PMSF protease inhibitor and homogenized using a pellet pestle. After centrifuging twice for 10 min, 5000× *g* at 4 °C, supernatants were freshly made prior to detecting the content of MCT and LPS in the feces. The supernatant was taken to detect MCT and LPS content by enzyme-linked immunosorbent assay using human trypsin (TPS) ELISA Assay Kit and human lipopolysaccharide (LPS) ELISA Assay Kit (Shanghai Tongwei, Shanghai, China) according to the manufacturer’s instructions.

### 2.8. Statistical Analysis

Bioinformatic analysis of the gut microbiota was carried out using the Majorbio Cloud platform (https://cloud.majorbio.com, accessed on 15 September 2023). The similarity among the microbial communities in different samples was determined by principal coordinate analysis (PCoA) based on Bray–Curtis dissimilarity. For metabolomics analysis, multivariate statistical analysis was performed using the ropls R package from Bioconductor (Version 1.6.2, http://bioconductor.org/packages/release/bioc/html/ropls.html, accessed on 15 September 2023) on the Majorbio Cloud Platform. Variable importance in the projection (VIP) values was calculated using an orthogonal projection to latent structures discriminant analysis (OPLS–DA) model. *p* values were estimated using a paired-sample Student’s *t*-test for single-dimensional statistical analysis. SPSS 17.0 (Chicago, IL, USA) and Origin 2018 (Origin Lab, Northampton, MA, USA) were used to analyze the data, and the data are expressed as means ± standard deviation (*n* = 12).

## 3. Results

### 3.1. Effects on Intestinal Lipopolysaccharides (LPSs), Mast Cell Trypsin (MCT), and Intestinal Microbiota Composition

Figure 1A illustrates the study design, encompassing 12 participants who lived in a confined environment for 14 days while adhering to a shift-work system. Analysis of fecal samples revealed that the concentrations of MCT (*p* < 0.01) and LPSs (*p* < 0.05) were significantly higher at the experiment’s conclusion (W3 point) compared to the beginning (W1 point) (Figure 1B,C). It indicated that living and working in a confined environment leads to changes in the intestinal environment that may affect the intestinal inflammatory balance and tissue sensitivity [19,20]. Gut microbiota analysis indicated that, in comparison to the study’s W1 point, the α-diversity was markedly decreased after 7 days of confinement (W2 point). However, no significant difference was observed after an additional 7 days (totaling 14 days) in the confined environment (W3 point) when compared to the initial W1 point (Figure 1D).

Figure 1E,F depict the impact of the experiment on the gut microbiota composition at the phylum and genus levels, respectively. At the phylum level, the gut microbiota on day 1 (W1 point) was predominantly (>99%) *Firmicutes* and *Bacteroidota*, while on day 14 (W3 point), it was primarily composed of *Firmicutes*, *Actinobacteriota*, and *Proteobacteria*. At the genus level, the gut microbiota of the W1 point was primarily composed of *Faecalibacterium*, *Bacteroides*, *Megamonas*, *Blautia*, and other bacterial genera, whereas that of the W3 point was mainly composed of *Blautia*, *Bifidobacterium*, *Collinsella*, *Agathobacter*, and other bacterial genera. These findings suggest that the confined environment significantly altered the gut microbiota composition of the participants.

### 3.2. Analysis of Differences in the Gut Microbiota

**Figure 2:**
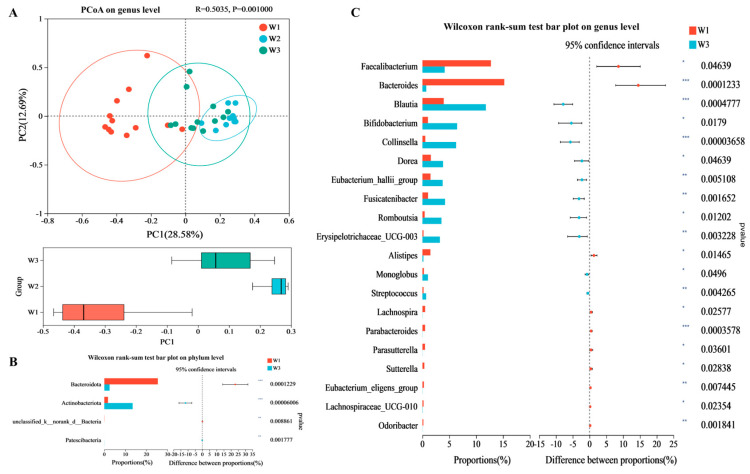
Analysis of differences in the gut microbiota. (**A**) PCoA (principal coordinate analysis) of gut microbiota based on Bray–Curtis dissimilarity. (**B**) Phylotypes are significantly different between W1 and W3 time points on the phylum level. (**C**) Phylotypes are significantly different between W1 and W3 time points on the genus level. The significance of the differences between the data was assessed using one-way ANOVA by Dunnett’s analysis, * *p* < 0.05, ** *p* < 0.01, *** *p* < 0.001.

Principal coordinate analysis indicated that the gut microbiota structure at the W3 point was significantly distinct from the W1 point at the genus level (*p* = 0.001), with reduced dispersion (Figure 2A). This difference could be due to the participants maintaining a sleep schedule at the W1 point. A significance test comparing the gut microbiota between the W1 and W3 points demonstrated a substantial difference at the phylum level (*p* < 0.001). Specifically, compared with the W1 point, the relative abundance of *Bacteroidota* was significantly decreased and that of *Actinobacteriota* was significantly increased at the W3 point (Figure 2B). Among the top 20 genera, the relative abundance of *Faecalibacterium*, *Bacteroides*, *Alistipes,* and other genera was significantly decreased, and that of *Blautia*, *Bifidobacterium*, *Collinsella*, *Dorea*, *Eubacterium_hallii_group*, and *Fusicatenibacter* was significantly increased at the W3 point compared with the W1 point (Figure 2C).

### 3.3. Non-Targeted Metabolomics Analysis of Fecal Samples

**Figure 3:**
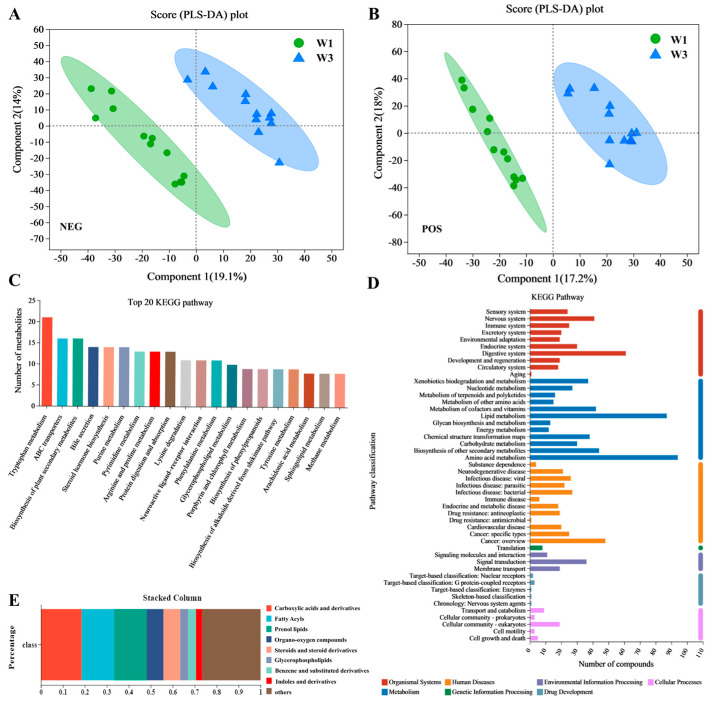
Non-targeted metabolomics analysis of fecal samples. (**A**) PLS-DA of metabolomics data for different fecal samples under NEG model. (**B**) PLS-DA of metabolomics data for different fecal samples under POS model. (**C**) Top 20 KEGG pathways of metabolites. (**D**) KEGG pathway classification: metabolites detected and annotated. (**E**) HMDB classification of annotated metabolites.

Fecal samples from the participants (W1 and W3) were analyzed using non-target metabolomics techniques. Following data pre-processing, 1490 metabolites were identified in positive-ion mode and 898 in negative-ion mode. Analysis of samples using partial least squares discriminant analysis (PLS-DA) revealed significant differences in metabolomic profiles between the W3 and W1 points (Figure 3A,B).

As shown in Figure 3C, all metabolites were annotated to the Kyoto Encyclopedia of Genes and Genomes (KEGG) pathways, indicating that pathways related to tryptophan metabolism, ATP-binding cassette transporters, bile secretion, and steroid hormone biosynthesis were notably more active than other pathways. Figure 3D illustrates that metabolites were predominantly linked to KEGG metabolic pathways, including those for metabolism, human diseases, and organismal systems, with the highest number of metabolites annotated in amino acid metabolism (94), lipid metabolism (87), and digestive system (61) pathways. According to the Human Metabolome Database (HMDB) classification, at the class level, metabolites with a relative abundance exceeding 1% were predominantly carboxylic acids and derivatives (18.50%), fatty acyls (14.84%), prenol lipids (14.84%), and organo-oxygen compounds (7.64%), as shown in Figure 3E.

### 3.4. Differential Metabolite Analysis

**Figure 4:**
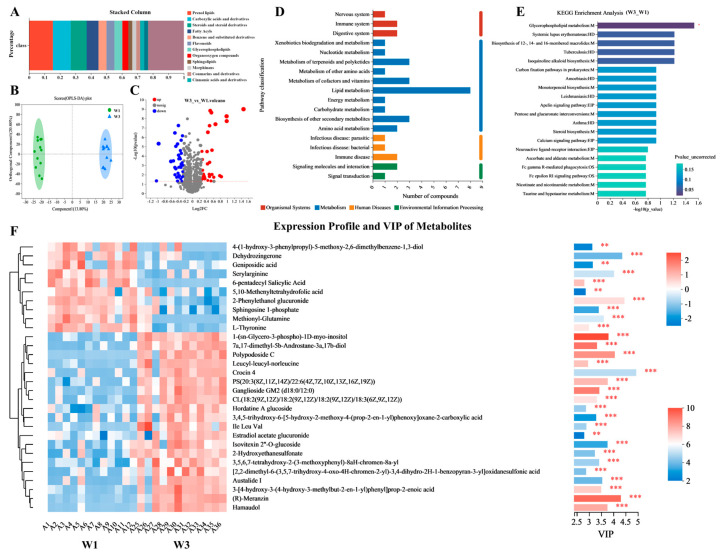
Differential metabolites analysis in metabolic set of W3_W1. (**A**) HMDB classification of differential metabolites. (**B**) PLS-DA of differential metabolites in fecal samples. (**C**) Volcano plot of differential metabolites. (**D**) KEGG pathway classification of differential metabolites. (**E**) KEGG enrichment analysis of metabolic pathways identified between W3 and W1. (**F**) Metabolite clustering heatmap analysis and variable importance in projection (VIP) scores of differential metabolites between W3 and W1. Selected metabolites (VIP top 30) were those with VIP > 1.0. VIP score was based on OPLS-DA model. Significant differences were compared with each two groups (** *p* < 0.01, *** *p* < 0.001).

A differential metabolic set (W3_W1) was established according to specific criteria (fold change [FC] > 1.2, variable importance in projection [VIP] > 1, and *p* < 0.05), resulting in the identification of 122 differential metabolites. Analysis using HMDB indicated that among these metabolites, those with a relative abundance exceeding 1% were predominantly prenol lipids (15.38%), carboxylic acids and their derivatives (11.54%), steroids and steroid derivatives (10.26%), and fatty acyls (7.69%), as illustrated in Figure 4A. The orthogonal projections to latent structures discriminant analysis (OPLS-DA) results demonstrated that the differential metabolites at the W3 point were clearly differentiated from those at the W1 point, suggesting a substantial impact of the experimental environment on participants’ metabolic profiles, as depicted in Figure 4B. As shown in Figure 4C, volcanic map analysis showed that compared with the W1 point, the W3 point had 36 upregulated metabolites and 40 downregulated metabolites. The KEGG annotation results indicated that the majority of differential metabolites were associated with lipid metabolism pathways, as shown in Figure 4D. Further KEGG enrichment analysis revealed significant enrichment in glycerophospholipid metabolism among the differential metabolites (*p* < 0.05), as indicated in Figure 4E.

Figure 4F presents a heatmap analysis of the top 30 differential metabolites sorted by VIP, highlighting significant differences in the metabolite profiles between the W3 and W1 points. Several metabolites, including 7α,17-dimethyl-5β-androstane-3α,17β-diol, 1-(sn-glycero-3-phospho)-1D-myo-inositol, crocin, and histidinyl hydroxyproline, were notably upregulated at the W3 point, whereas others like sphingosine 1-phosphate, 5,10-methenyltetrahydrofolic acid, 7-dehydrodesmosterol, 16β-hydroxyestrone, and methionylglutamine were found to be significantly downregulated at the W1 point.

### 3.5. Screening of Metabolic Markers

**Figure 5:**
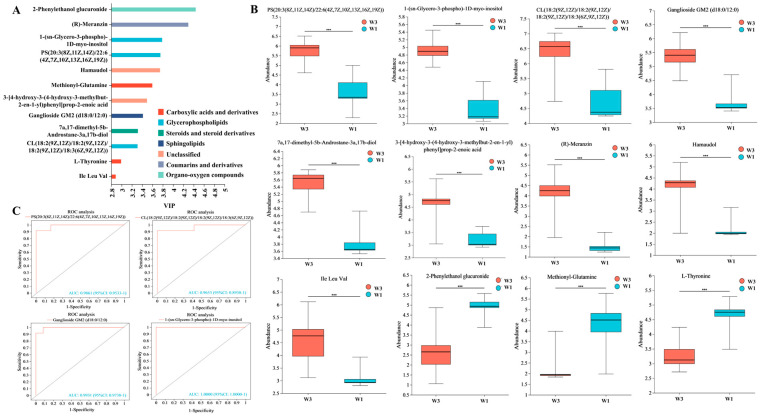
Metabolic markers analysis in confined environment. (**A**) HMDB classification of top 25 differentially expressed metabolites was sorted by VIP values. (**B**) Comparative analysis of the relative content of metabolites (ROC > 0.95). (**C**) ROC curves of four lipid metabolites upregulated in W3 time point. Significant differences were compared with each two groups (*** *p* < 0.001).

The top 25 differentially expressed metabolites, sorted by VIP values and assessed for biomarker potential, are depicted in Figure 5. Analysis using the receiver operating characteristic (ROC) curve indicated that 12 metabolites, with VIP values between 2.88 and 4.44, exhibited areas under the curve exceeding 0.95. Classification by HMDB revealed that these metabolites were predominantly glycerophospholipids, carboxylic acids and their derivatives, and sphingolipids, as illustrated in Figure 5A. Figure 5B presents a comparative analysis of the expression levels of the 12 identified metabolites, highlighting the changes occurring after participants’ exposure to the confined environment. It demonstrates that nine metabolites exhibited upregulation, while three showed downregulation. The KEGG pathway annotation suggests that lipid metabolic pathways were significantly active among participants throughout the experiment. Consequently, ROC curves were constructed for four specific lipid metabolites: 1-(sn-glycero-3-phospho)-1D-myo-inositol, phosphatidylserines (20:3(8Z,11Z,14Z)/22:6(4Z,7Z,10Z,13Z,16Z,19Z)), ganglioside monosialic 2 (d18:0/12:0), and cardiolipins (18:2(9Z,12Z)/18:2(9Z,12Z)/18:2(9Z,12Z)/18:3(6Z,9Z,12Z)). Figure 5C illustrates that these four lipid metabolites could potentially serve as biomarkers for metabolic processes that are upregulated in individuals working in confined environments.

### 3.6. Association between Gut Microbiota and Metabolome

**Figure 6:**
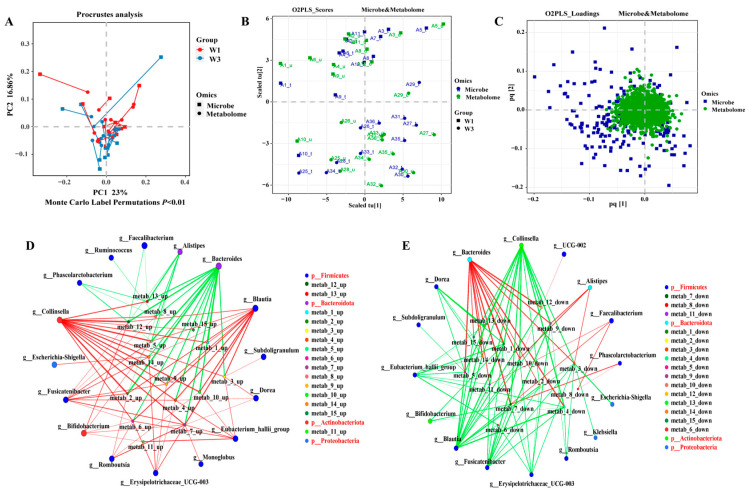
Association between gut microbiota and metabolome. (**A**) Procrustes analysis of gut microbiota and metabolome. (**B**,**C**) O2PLS analysis of gut microbiota and metabolome. (**D**) Correlation network analysis of gut microbiota (top 15 of relative abundance) with upregulated differential metabolites (top 15 VIP). (**E**) Correlation network analysis of gut microbiota (top 15 of relative abundance) with downregulated differential metabolites (top 15 VIP).

Procrustes analysis, a technique in multivariate statistics, is utilized for the comparative analysis of traits, aiming to determine the best alignment between two sets of geometric shapes through rotation, translation, reflection, and scaling. Accordingly, this method was applied to assess the correlation between the spatial arrangement of participants’ gut microbiota and fecal metabolites, as well as to explore the similarities and differences between the microbiomes and metabolomes. The findings revealed a significant correlation between the diversity of the gut microbiota and the metabolite levels (*p* < 0.01), suggesting a robust association between gut microbiota composition and metabolite profiles (Figure 6A). Additionally, a two-way orthogonal partial least squares (O2PLS) analysis confirmed a strong correlation between gut microbiota composition and the metabolome (Figure 6B,C).

From the differential metabolic set W3_W1, the top 15 upregulated and downregulated metabolites were identified (Table 1) and subjected to an association network analysis with the gut microbiota. Figure 6D,E illustrate that, at the genus level, the gut microbiota displayed a complex network of interactions with the selected differential metabolites. The upregulated differential metabolites demonstrated a strong positive correlation with the genera *Collinsella*, *Blautia*, and *Fusicatenibacter*, while showing a strong negative correlation with *Bacteroides*. Conversely, the downregulated differential metabolites exhibited opposite correlation patterns with the aforementioned gut microbial genera.

### 3.7. Significance Analysis of the Association of the Gut Microbiota with Metabolites

**Figure 7:**
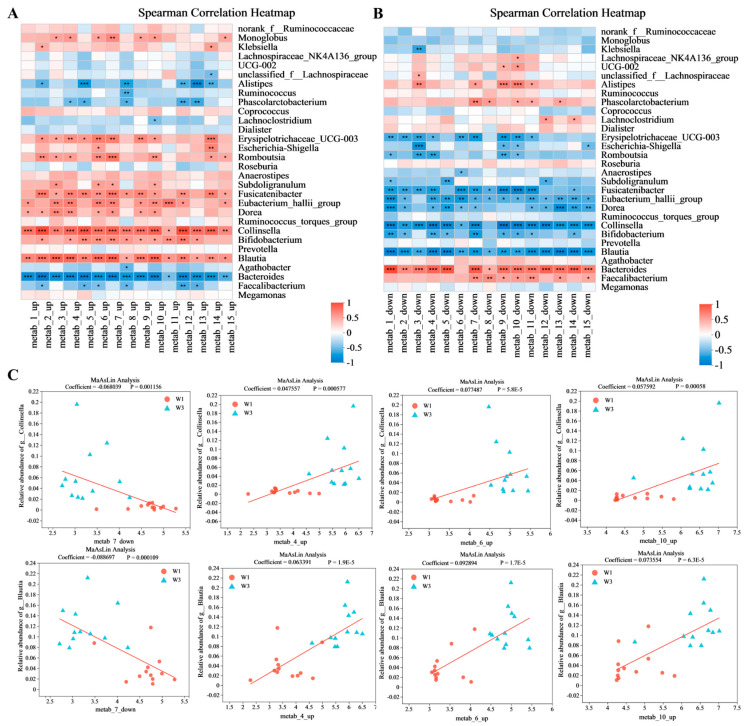
Significance analysis of association of gut microbiota with metabolites. (**A**) Correlation heatmap of gut microbial and top 15 (VIP) upregulated metabolites. (**B**) Correlation heatmap of gut microbial and top 15 (VIP) downregulated metabolites. (**C**) MaAsLin analysis of gut microbiota and differential metabolites. Significant differences were compared with each two groups (* *p* < 0.05, ** *p* < 0.01, *** *p* < 0.001).

Figure 7 presents a correlation heatmap illustrating the relationships between the top 30 gut microbial genera by relative abundance and the top 15 metabolites by VIP score. The heatmap analysis demonstrated significant correlations between metabolites and the gut microbiota, irrespective of the metabolites’ regulation status. Specifically, as depicted in Figure 7A, *Bacteroides* and *Alistipes* were significantly negatively associated with upregulated metabolites, while *Collinsella*, *Blautia*, and *Fusicatenibacter* exhibited significant positive associations with these metabolites. Conversely, the correlations between the gut microbiota and downregulated metabolites mirrored the inverse of those observed with upregulated metabolites, with the strongest effects noted for *Collinsella*, *Blautia*, and *Bacteroides*. To substantiate the observed correlations between the gut microbiota and four potential lipid metabolism biomarkers, a MaAsLin-based correlation analysis was conducted, with results detailed in Figure 7C. The findings indicated that following the participants’ exposure to the confined environment, the enrichment of *Collinsella*, *Blautia*, and other genera within their gut microbiota was strongly correlated with these four biomarkers.

## 4. Discussion

In this study, the participants performed experiments in frequent shifts in a strictly confined environment. Multi-omics techniques were employed to assess alterations and correlations within the gut microbiota and metabolites. In confined workspaces, such as deep-sea scientific expeditions, workers must often perform frequent shifts, which perturbs dietary, sleep, and other rhythms, thereby affecting the gut microbiota and its metabolic characteristics. Notably, night shifts can disrupt circadian rhythms, diminish melatonin production, and heighten the risk of metabolic disorders, including cardiovascular diseases [21,22,23]. Shift workers, especially those working night shifts, are frequently exposed to artificial light and experience significant changes in their sleep patterns and eating times. This can lead to the desynchronization of the central and peripheral circadian clocks and disrupt the rhythmic changes of the gut microbiota, thereby contributing to metabolic diseases [24,25]. Furthermore, individuals in confined environments experience greater social isolation compared to those in normal environments, potentially intensifying appetite loss and mood disturbances. Additionally, research by Li et al. has indicated that in confined environments, the gut microbiota’s composition and functionality significantly influence workers’ moods [26].

In healthy individuals, up to 60% of the gut microbiota oscillates rhythmically [27]. Metabolic abnormalities associated with rhythm disorders are closely related to perturbations of oscillations in the gut microbiota and its products [25,28]. In this study, the gut microbiota structure of the participants was significantly different after they were exposed to the confined environment, contrasting with their pre-exposure state. In particular, there was a significant rise in the relative abundance of *Bacteroides* and *Actinobacteriota*. This suggests that the participants’ gut microbiota composition and oscillation patterns were disrupted, potentially affecting their circadian rhythm regulation [29,30]. Shift work in confined environments, leading to sleep deprivation, can disrupt the rhythmic oscillations of *Bacteroides*, a finding that corroborates Li et al.’s research [31]. Additionally, sleep deprivation can weaken our immune function through changes in the gut microbiota [32,33,34]. Furthermore, disorders of gene expression involved in liver rhythm regulation, such as those of *Bmal1* and *Clock*, were observed in high-energy diet models and are related to *Bacteroides* [35].

Confined environments can cause constant psychological stress and thus significantly affect mood [36]. Sustained stress can also trigger the hypothalamus–pituitary–adrenal axis, which affects the gut microbiota by reducing the relative abundance of *Lactobacillus* and *Bacteroides* [37]. Hao et al. showed that during the 1-year “Lunar Palace 365” project, the presence of potential probiotics such as *Bacteroides uniformis* and *Roseburia inulinivorans* were positively correlated with positive emotions in participants [38]. Lu et al. indicate that the working environment of underground tunnels can cause significant changes in the gut microbiota, which may be related to the workers’ psychological stress and emotional abnormalities [39].

*Bacteroides*, *Roseburia*, and other genera are able to metabolize carbohydrates, so significant changes in their abundance may be related to the perturbation of people’s dietary rhythms in confined environments [40]. *Bacteroides* regulate a depressed mood by influencing bidirectional communication via the brain–gut axis through tryptophan metabolic pathways and neurotransmitter conduction, but this function is species-dependent [41]. Furthermore, in this study, the relative abundance of *Faecalibacterium* was found to be significantly reduced in participants after they had lived in a confined environment. As *Faecalibacterium* is directly proportional to sleep quality in patients with bipolar disorder, increasing the abundance of *Faecalibacterium* improved sleep quality [42]. Grosicki et al. showed that in young, healthy individuals, the relative abundance of *Blautia* and *Ruminococcus* in the gut microbiota was inversely proportional to sleep quality, while that of *Bacteroidetes* was positively associated with sleep quality [43].

Changes in the gut microbiota can significantly impact the intestinal microenvironment, inflammatory state, and metabolites. In this study, after living and working in a confined environment, the LPS concentrations in the fecal matter of participants were significantly increased, which may be related to the increased abundance of the genus *Collinsella* [44]. *Collinsella*, a member of the *Actinobacteriota* phylum, is closely associated with various diseases, including diabetes and nonalcoholic steatohepatitis [45,46]. Moreover, Kalinkovich et al. found that *Collinsella* has pro-inflammatory effects, such as its capability of epitope mimicry and enhancing cell apoptosis [47].

Intestinal metabolites are shaped by numerous factors, including diet, gut microbiota, and host metabolic processes. Our metabolomic analyses revealed that frequent shift work within a confined environment resulted in a substantial upregulation of lipid metabolic pathways and elevated levels of various lipid metabolites, potentially linked to disrupted rhythms and sleep deprivation [48]. Lipid metabolites are essential for various cellular functions, including biofilm formation, cell signaling, and protein–chromosome interactions, and are significantly correlated with sleep, mood, and circadian rhythm regulation [49]. Furthermore, the synthesis and metabolic processes of lipid metabolites, including glycerophospholipids, are under complex and stringent temporal regulation in cells such as retinal cells and fibroblasts [50,51]. Moreover, fluctuations in gut microbial abundance also influence the composition of lipid metabolites. For example, sphingolipids are the main lipid components of the exosomes of *Bacteroides*, which are involved in the digestion of carbohydrates [52]. The relative abundance of *Bacteroides* decreased significantly in the participants in this study after they had lived in the confined environment, resulting in a decrease in the content of sphingolipids in their fecal samples compared with before the experiment.

## 5. Conclusions

We examined the effects of confined environments on participants’ gut microbiota and metabolism under a strict shift work system. Studies have shown that shift work in a confined environment can seriously affect the structure and composition of gut microbiota, and can significantly increase the abundance of *Collinsella*, *Blautia,* and other genera of bacteria. At the same time, it can also significantly increase the content of LPS and MCT in feces. Metabolomics studies showed that after the experiment, the activity of lipid metabolism in the intestinal metabolites of the subjects increased significantly, and the changes in the intestinal metabolites had a strong correlation with the changes in the gut microbiota. We screened four biomarkers associated with confined environment experiments from lipid metabolites and found that these metabolic markers were positively correlated with the enrichment of *Collinsella*, *Blautia,* and other genera of bacteria. This suggests that gut microbiota may be a potential therapeutic target for mitigating the health effects of confined environments such as deep sea and aerospace.

Probiotics and dietary interventions can effectively improve gut microbiota dysbiosis, which may potentially alleviate the effects of confined spaces. So, future studies will explore the impact of dietary interventions on the effects of confined environments and validate the efficacy of the identified potential biomarkers.

## Figures and Tables

**Table 1:** The information of the top 30 metabolites (sorted by VIP).

Metabolites	Code	VIP_PLSDA	*p*	FC (W3/W1)
2-Phenylethanol glucuronide	metab_1_down	3.945	2.25 × 10^−7^	0.536
Dehydrozingerone	metab_2_down	3.8852	0.0001184	0.2979
Serylarginine	metab_3_down	3.4511	7.42 × 10^−6^	0.6066
Methionyl-Glutamine	metab_4_down	3.4181	4.80 × 10^−6^	0.5118
Sphingosine 1-phosphate	metab_5_down	3.1949	0.0003718	0.6538
Geniposidic acid	metab_6_down	2.8451	0.00153	0.6132
L-Thyronine	metab_7_down	2.7952	3.44 × 10^−7^	0.7068
4-(1-hydroxy-3-phenylpropyl)-5-methoxy-2,6-dimethylbenzene-1,3-diol	metab_8_down	2.7574	0.003052	0.5893
6-pentadecyl Salicylic Acid	metab_9_down	2.655	5.62 × 10^−8^	0.7579
CP 47,497-C8-homolog C-8-hydroxy metabolite	metab_10_down	2.6112	5.17 × 10^−6^	0.7451
Tiazuril	metab_11_down	2.6021	9.18 × 10^−6^	0.7204
Repaglinide aromatic amine	metab_12_down	2.5741	0.002264	0.7198
5,10-Methenyltetrahydrofolic acid	metab_13_down	2.5588	0.006257	0.673
Humulenol II	metab_14_down	2.5371	0.001422	0.7852
Met Ile Lys His	metab_15_down	2.5225	8.10 × 10^−5^	0.7595
Crocin 4	metab_1_up	4.6252	1.18 × 10^−5^	3.2293
(R)-Meranzin	metab_2_up	4.0333	1.01 × 10^−9^	2.7535
Polypodoside C	metab_3_up	3.8511	5.77 × 10^−9^	1.9694
PS(20:3(8Z,11Z,14Z)/22:6(4Z,7Z,10Z,13Z,16Z,19Z))	metab_4_up	3.5563	4.06 × 10^−8^	1.5789
Hamaudol	metab_5_up	3.5003	1.89 × 10^−8^	1.9738
1-(sn-Glycero-3-phospho)-1D-myo-inositol	metab_6_up	3.3895	2.29 × 10^−10^	1.4525
Ganglioside GM2 (d18:0/12:0)	metab_7_up	3.2341	2.35 × 10^−9^	1.457
Isovitexin 2″-O-glucoside	metab_8_up	3.2274	0.0003862	1.8025
7a,17-dimethyl-5b-Androstane-3a,17b-diol	metab_9_up	3.2009	1.46 × 10^−9^	1.4234
CL(18:2(9Z,12Z)/18:2(9Z,12Z)/18:2(9Z,12Z)/18:3(6Z,9Z,12Z))	metab_10_up	3.1481	2.92 × 10^−7^	1.3588
3,4,5-trihydroxy-6-[5-hydroxy-2-methoxy-4-(prop-2-en-1-yl)phenoxy]oxane-2-carboxylic acid	metab_11_up	3.1198	0.0008611	1.7527
3-[4-hydroxy-3-(4-hydroxy-3-methylbut-2-en-1-yl)phenyl]prop-2-enoic acid	metab_12_up	3.1166	9.90 × 10^−8^	1.4571
Austalide I	metab_13_up	3.036	0.0001106	1.4601
3,5,6,7-tetrahydroxy-2-(3-methoxyphenyl)-8aH-chromen-8a-yl	metab_14_up	2.9157	2.55 × 10^−5^	1.3227
Leucyl-leucyl-norleucine	metab_15_up	2.8408	5.84 × 10^−8^	1.4052

## Data Availability

The data presented in the study are deposited in the BioProject database, accession numbers PRJNA1033593, and PRJNA1042836.
